# Publication of Medical Society Proceedings

**Published:** 1881-05

**Authors:** 


### EDITORIAL DEPARTMENT.

"If thou hast truth to utter, speak;
And leave the rest to God."

PUBLICATION OF MEDICAL SOCIETY PROCEEDINGS.—It would tend to the advantage of medical science, if the societies that still adhere to the plan of publishing the papers read at their annual meetings, in book or pamphlet form, could be induced to alter their programme, or laws, somewhat. Those published proceedings rarely exceed a few hundred copies, and that small number even, is usually so distributed as to add but little to either the advancement of science or the reputation of the authors of valuable papers. If the societies acting thus would merely publish their *official* proceedings, and the less valuable or valueless effusions with which they are all, more or less, afflicted on anniversary occasions, and permit the valuable contributions to go into medical journals of considerable circulation, our science would be greatly enriched thereby. Take our State Medical Society—the Medical and Chirurgical Faculty, for example, before which several really valuable papers were read at its recent meeting in this city, and yet, owing to a law of the Society on the subject, *all* papers read before the Society become the property of the Society and are published, or not, in their annual volume of transactions, as the committee on publication may elect. Application was made to the author of one of these papers for the privilege of putting his article in print, a few days prior to the annual meeting, so that it might appear in this journal soon after the adjournment of the Society, and it would thus have been read by thousands ere this, who will now have to await the publication of the "proceedings," and it may be some time yet before it will be seen by the limited number of readers to which the annual volume finds its way! all because of the obnoxious prohibitory law alluded to above. We are glad to say such laws have been repealed, or rendered inoperative, in some of the state societies, and it were well if it were done in all of them. We have endeavored from the inception of this enterprise to procure carefully prepared papers for its pages, believing that, as far as possible, such should alone be published; and but for the prohibition of our own, and a few other societies, we would be able to furnish our thousands of readers with really valuable information which will never be known, in some instances at least, beyond the narrow limits of a few hundred members and half that number of exchanges.

